# Exogenous Gibberellic Acid (GA_3_) Enhances Mango Fruit Quality by Regulating Resource-Related Metabolic Pathways

**DOI:** 10.3390/plants15030482

**Published:** 2026-02-04

**Authors:** Lina Zhai, Lixia Wang, Ghulam Abbas Shah, Tao Jing, Hafiz Faiq Bakhat, Yan Zhao, Yingdui He

**Affiliations:** 1 College of Tropical Agriculture and Forestry, Hainan University, Haikou 570228, China; 23220951320019@hainanu.edu.cn; 2 Institute of Tropical Bioscience and Biotechnology, Sanya Research Institute, Chinese Academy of Tropical Agricultural Sciences, Haikou 571101, China; wanglixia@catasitbb.cn (L.W.); shahga@uaar.edu.pk (G.A.S.); jingtao@catasitbb.cn (T.J.); faiqsiddique@ciitvehari.edu.pk (H.F.B.); 3 Department of Agronomy, PMAS-Arid Agriculture University, Rawalpindi 46300, Pakistan; 4 Department of Environmental Sciences, COMSATS University Islamabad, Vehari Campus, Islamabad 61100, Pakistan

**Keywords:** metabolomics, plant growth regulator, postharvest physiology, sensory quality, tropical fruit

## Abstract

Efficient resource allocation during fruit expansion and ripening is critical for enhancing mango (*Mangifera indica* L.) productivity and fruit quality. A study was conducted to quantify the effects of foliar-applied GA_3_ at concentrations of 0 (control), 50 (GA50), 100 (GA100) and 200 (GA200) mg L^−1^, applied at 15, 25 and 35 days after full bloom, on fruit physiochemical attributes during the fruit expansion and ripening phases. In addition, metabolic profiling and pathway analysis were conducted after fruit ripening. Compared with the control, GA_3_ application at 50, 100, and 200 mg L^−1^ increased fruit length by 8, 12, and 14%, and fruit diameter by 5, 11, and 14%, respectively. The mean single-fruit weight was increased by 5–11% at physiological maturity. During the fruit expansion phase, GA_3_ treatment decreased starch and total acidity by up to 11% and 29%, respectively, while increasing the soluble sugar content by 21%. Furthermore, enhanced antioxidant enzyme activities (SOD, POD, and CAT), accompanied by a reduction in malondialdehyde (MDA) contents in leaves, were observed. At the ripening stage, GA_3_-treated fruits exhibited lower weight loss, higher firmness, more uniform color development, and reduced disease incidence, although vitamin C content and total soluble solids declined. PCA analysis identified GA100 as the optimal treatment. Metabolomics analysis revealed 287 differentially regulated metabolites between GA100 and the control. Sweet, fruity, and floral compounds were upregulated, whereas terpenoids and aldehydes were downregulated. KEGG pathway analysis indicated that GA100 modulated key resource-related metabolic pathways, including nitrogen, carbon and energy metabolism, thereby promoting efficient resource allocation toward fruit growth, quality, and aroma development. Overall, preharvest foliar application of GA_3_, particularly at a concentration of 100 mg L^−1^ (GA100), markedly improved mango fruit growth and quality but tended to simplify the aroma profiles by favoring ester production over complex terpenoid-derived notes.

## 1. Introduction

Mango (*Mangifera indica* L.), often called the “King of Fruits”, is the second most abundantly produced tropical fruit globally after banana. Its cultivation extends from tropical to subtropical regions of the world, with annual global production of 55 million metric tons and trade value of USD 1 billion [1]. India and China are the top producers of mango, contributing around 60% of its global production [1,2]. Mango cultivation in China is concentrated in southern provinces, such as Hainan, Guangdong, Guangxi, and Yunnan, where the climate is most favorable [3]. In addition to providing economic returns to the farmers, mangoes offer consumers a highly nutritious fruit rich in macro- and micronutrients, carbohydrates, fiber, protein, vitamins, minerals, and healthy fat [4]. Tainong 1 is a major cultivated variety, accounting for 45% of total mango cultivation in Hainan Province in China due to its unique flavor and high nutritional value. In addition, it has smooth flesh, fewer fibers and abundant juice [5]. The thick golden peel protects the fruit from storage and transportation damage. Overall, mango is highly perishable, with postharvest losses up to 30% in China, primarily caused by rapid biochemical and physiological changes during the ripening phase [6]. These transformations include cell wall breakdown, starch-to-sugar conversion, pigment changes, and increased respiration, which directly influence texture, flavor, and nutritional quality, thereby highlighting the need for precise ripening management to reduce postharvest losses and enhance marketability [7,8].

A wide range of postharvest techniques, such as edible coatings, chemical treatments, temperature management and biocontrol agents, have been used to reduce mango losses and maintain its nutritional value [9,10,11,12]. However, the adoption of these strategies remains limited, largely due to high costs, lack of awareness, infrastructure gaps, and practical constraints under field conditions. In contrast, preharvest strategies, such as fruit bagging and the use of growth regulators during the fruit expansion phase, have shown considerable effects in reducing postharvest losses up to 70% and extending shelf life by up to 15 days [13,14]. Among these, the preharvest application of gibberellic acid (GA_3_) has received particular attention, as it can extend the shelf life of mango fruit by up to 18 days, reduce fruit weight losses by 32%, and maintain high fruit firmness to 18.4 N compared with untreated fruit [15,16]. In addition, fruit size, fruit weight, soluble solids and titratable acidity were also increased [17,18]. However, these changes are dose-dependent, as low to moderate doses (20–100 mg L^−1^) enhance fruit size, firmness, chlorophyll retention, and resistance to senescence, whereas higher doses (200–400 mg L^−1^) excessively delay ripening and interfered with flavor development in mango [15,16]. Henceforth, understanding how various GA_3_ doses affect metabolic and physiological processes in mango is crucial for optimizing fruit quality and flavor over an extended period. Fruit formation and development make up a complex and nutrient-intensive process that involve metabolic reprogramming within the plant. During this phase, the role of leaves is not limited to serving as a source of photosynthates but also includes acting as a critical supplier of mineral nutrients. These processes can trigger the production of reactive oxygen species (ROS), which interact with plant hormones and affect developmental pathways [19]. However, GA_3_-induced suppression of ROS has been reported, and its role in leaves during the fruit expansion stage, when the plant undergoes tremendous metabolic changes, remains elusive.

Preharvest application of GA_3_ decreased concentrations of various monoterpenes in rambutan and longan due to the downregulation of terpene biosynthetic activity during the ripening phase [20,21]. The formation of aldehydes in rambutan was diminished due to the inhibition of lipoxygenase activity and slow membrane turnover [21]. Ester production in grapes and rambutan was strongly suppressed due to reduced ethylene biosynthesis and alcohol acyltransferase activity, resulting in delayed aroma development [21,22]. In addition, the levels of ketones and minor aromatic hydrocarbons were also reduced. Overall, GA_3_ application modulates key carbon, nitrogen, lipid, and secondary metabolite-related pathways, thereby suppressing the biosynthesis of key aroma-related volatiles and affecting the overall aromatic quality of mango fruit. Moreover, it delays the accumulation of ripening-related metabolites in the fruit. Despite the recognized role of GA_3_ in modulating fruit development and delaying ripening, there is limited comprehensive understanding of how different preharvest application rates of GA_3_ affect the full spectrum of mango fruit physicochemical attributes and underlying resource-related metabolic pathways during fruit development and ripening phases. Therefore, this study aimed to address these gaps by analyzing the effects of the preharvest application of various doses of GA_3_ on changes in the fruit physicochemical attributes of “Tainong 1” mango (fruit size, weight, starch, soluble solids and total acidity as well as leaves’ antioxidant activities) during the expansion phase and flavor-associated metabolites and resource-related metabolic pathways during the ripening phase. We hypothesized that preharvest application of GA_3_ at a medium concentration of 100 mg L^−1^ would significantly alter the fruit’s physicochemical attributes during the fruit expansion phase and modulate flavor-associated metabolites and resource-related metabolic pathways in mango. This work reports preharvest management strategies designed to enhance value-added processing of mango fruit during the ripening phase, thereby optimizing the economic and nutritional potential of mango fruit in tropical regions of China.

## 2. Materials and Methods

### 2.1. Experimental Site

The experiment was carried out at mango orchard located in Qianjia Town, Le Dong County, Hainan Province, China (latitude 18.56924° N and longitude 109.08203° E). The orchard is located 166 m above sea level. The climate of the region is tropical monsoon with mean temperatures ranging from 12.4 °C (minimum) to 38.2 °C (maximum). The region has total rainfall of 1987.7 mm and total sunshine hours 1943.8 h [23]. The experimental soil is classified as sandy loam, with a pH of 5.51, organic matter content of 11.7 g kg^−1^, total nitrogen content of 1.4 g kg^−1^, alkali hydrolysable nitrogen content of 103.62 mg kg^−1^, total phosphorus content of 32.01 mg kg^−1^, available phosphorus content of 23.04 mg kg^−1^, total potassium content of 316.80 mg kg^−1^ and available potassium content of 179.80 mg kg^−1^ in the 0–45 cm soil layer.

### 2.2. Experimental Design and Treatments

The experimental orchard comprised 200 eight-year-old mango trees of cultivar ‘Tai-nong 1’ grafted on the locally selected seedling rootstock ‘Hainan Tumang (海南土芒)’ (*Mangifera indica* L.). The orchard was planted with plant-to-plant and row-to-row spacing of 4 m and 4.5 m, leading to a tree density of 555 plants/ha. The experiment was conducted using a Randomized Complete Block Design (RCBD) with three replications (blocks) and four treatments. Each block had four experimental plots, and treatments were randomly assigned to one plot within each block. Each experimental plot comprised 9 mango trees, of which 3 trees of similar size, free from disease and pests, were selected for treatment application, while the remaining trees served as buffer plants. The entire experiment comprised 108 mango trees arranged in 12 experimental plots. During each application, treatments were applied to the entire tree. Each plot was considered the experimental unit and biological replicate. For data collection, five fruit-bearing branches on each tree positioned toward the east, west, south, north, and center were selected and labelled. Branches and fruits were considered as subsamples. These subsamples from each plot were combined to obtain composite samples. To prevent spray drift and treatment overlap, no two treated trees were adjacent within and among the plots. Treatments were: foliar application of gibberellic acid (GA_3_) at rates of 50, 100 and 200 mg L^−1^ along with control (foliar application of deionized water). The selected GA_3_ concentrations are within the range of commonly tested doses for enhancing fruit retention, yield and post-harvest quality of mango [24,25]. The GA_3_ was commercially purchased from Beijing Solab Technology Co., Ltd. (Beijing, China) and had 90% purity. A volume of 2 L of GA_3_ solution (treated) or deionized water (control) was applied per plant through knapsack sprayer, ensuring complete wetting of the leaf surfaces without causing dripping. Foliar application of treatments was performed at 15 (29 March 2025), 25 (8 April 2025) and 35 (18 April 2025) days after full bloom (DAFB) (14 March 2025). The onset of the cell expansion phase in mango fruit development typically occurs around 15 DAFB, with a transition from cell division to cell expansion by 25 DAFB, accompanied by rapid growth. By 35 DAFB, fruits exhibited active cell expansion, rapid weight gain and substantial starch accumulation, which aligns with the period of maximum cellular activity and increased responsiveness to GA_3_ [26]. All GA_3_ applications were started at 4:00 p.m. without the use of any surfactant [17]. The tree shape for the experimental year was formed through pruning the branches after the mango harvest of the previous year. Additionally, fruit thinning was conducted during the experimental period, retaining 1-2 fruits per panicle to promote nutrient balance in the fruits [27]. All other cultivation measures remained consistent across all treatment groups.

### 2.3. Measurement of Fruit Length and Diameter During Fruit Expansion Phase

Fruit length and diameter were measured using vernier calipers with 0.01 mm precision while the fruits were still attached to the tree 25 (8 April 2025), 35 (18 April 2025) and 45 (28 April 2025) days after full bloom. For each sampling event, 30 mango fruits were selected from five labelled branches on each treated tree for measurement of fruit length and diameter. Measurements were obtained from three treated trees per plot, resulting in 90 fruit observations per plot. These observations were treated as subsamples and averaged to obtain a single mean value per plot for statistical analysis. After 55 days following full bloom (8 May 2025), 30 similar-sized and disease-free fruits without significant damage were carefully removed from marked branches and used for the measurements of fruit fresh weight, fruit length, diameter and peduncle lengths.

### 2.4. Determination of Fruit Starch, Total Soluble Solids and Total Acidity During Fruit Expansion Phase

From a composite sample of 90 fruits per plot taken 55 days after full bloom (8 May 2025), 5 fruits were randomly selected, and their pulp was mixed to obtain a representative sample per treatment for the analyses of starch soluble solids and total acidity. Starch of fruit samples was measured through acid hydrolysis method, as described by Kaliyappan and Ramanathan [28], with some modifications. For this purpose, 0.1 g of mango pulp was homogenized with 1 mL of 80% ethanol through ball mill. The blend was then incubated in water bath for 20 min at 80 °C, cooled down and centrifuged at 8000 rpm for 10 min. The supernatant was discarded, and pellets were washed with ethanol and, gelatinized in water bath at 95 °C for 15 min. After cooling, the precipitate was mixed with 0.35 mL perchloric acid followed by incubation at 95 °C for 10 min with vortexing every 5 min. Lastly, 0.85 mL of water was added, and solution was centrifuged at 4000× *g* for 10 min. The supernatant was diluted 100-fold, and starch was measured via anthrone colorimetric method. The determination of total soluble solids and total acidity in fruits was conducted by homogenizing mango pulp with deionized water at a ratio of 1:50 (mass to volume), filtering through double-layered gauze, and then taking 1 mL of the filtrate to be measured using a sugar-acid analyzer [29] (PAL-BX, Atago Co., Tokyo, Japan).

### 2.5. Determination of Leaf Antioxidant Enzyme Activities During Fruit Expansion Phase

Ten mango leaves were randomly selected from each plant (2 leaves × 5 labeled branches) 55 days after full bloom (8 May 2025). Leaves from three sprayed plants per plot (30 leaves in total) were pooled to form a composite sample for the determination of antioxidant enzyme activities. Antioxidant enzyme activities of mango leaves might reach the peak around 55 days after full bloom, which coincides with the maxi-mum cell expansion activities in fruits at this stage, suggesting a physiological link between leaf antioxidant status and fruit development [30]. Therefore, this stage was selected for leaf sampling to analyze antioxidant enzymes activities. After sampling, all leaves were washed with deionized water. Subsequently, 0.2 g of the leaf sample was homogenized in an ice bath using 2 mL of 50 mM ice-cold phosphate buffer (PBS) with a pH of 7.4. Subsequently, the homogenate was centrifuged at 10,000 rpm and 4 °C to obtain the supernatants. The activities of antioxidant enzymes in leaf tissues, including superoxide dismutase (SOD), peroxidase (POD), catalase (CAT), were determined using ultraviolet spectrophotometric method. The SOD activity was determined based on its ability to inhibit photoreduction of nitroblue tetrazolium. Increase in absorbance due to guaiacol oxidation showed POD activity, while decomposition rate of hydrogen peroxide revealed CAT activity. In addition, MDA contents, an indicator of membrane damage due to ROS, were assessed using thiobarbituric acid (TBA) reaction.

### 2.6. Scanning Electron Microscopy of Fruit Pulp

Scanning electron microscopy of mango pulp was conducted 93 days after full bloom (DAFB), representing a uniform development stage commonly associated with physiological maturity of Tainong 1. At this stage, mango fruit growth (cell division and expansion) is largely completed; the fruit has attained near-maximum size and is considered suitable for harvest, with further changes considered predominantly biochemical rather than growth-related. Sampling 93 DAFB allowed for consistent evaluation of treatment effects. A small bean-sized piece of pulp from the equatorial region of mango fruit was taken and fixed in 2.5% glutaraldehyde purchased from Sorelbo, Beijing Sorelbo Technology Co., Ltd. (Beijing, China). Samples were pretreated according to the method described by Beirão-da-Costa et al. [31] and subsequently examined using a scanning electron microscope (SU8100, HITACHI, Tokyo, Japan) at a working voltage of 3.0 kV and a magnification of 500×.

### 2.7. Determination of Fruit Physicochemical Attributes During Fruit Ripening Phase

At physiological maturity (93 days after full bloom; 15 June 2025), fifteen mango fruits with consistent size, maturity, and without mechanical damage were hand-harvested from labelled branches of each tree. A total of 45 fruits per plot were collected from three treated trees and combined to form a composite sample. Five fruits were randomly selected from each composite sample for SEM analysis, while the remaining forty fruits per plot per replication were immediately stored in a preservation box. Each layer of mangoes was isolated and protected with pearl cotton padding, and placed at 25 °C and 60% relative humidity. Fruit physicochemical attributes were measured three times over a nine days storage period at three-day intervals. At each sampling point, five fruits were randomly selected from each replication for analysis. Weight loss was calculated through difference in weight at the beginning of storage period and weight at sampling day. Fruit firmness was determined using a texture analyzer (TMS-Pro, FTC Company, Washington, DC, USA) equipped with a flat cylindrical probe (P/5, 5-mm diameter). The probe was driven into the mango flesh at a constant speed, and the maximum force required to deform the tissue was recorded as firmness (N). Mango peel color was analyzed with CIELAB color space by using a chromameter (Konica Minolta CR-400/410, Osaka, Japan), as described by Oliveira et al. [32]. Both peel color differences and fruit firmness were measured twice in the middle portion of the fruit around its widest diameter, and average was taken. Fruit vitamin C was determined using the phosphomolybdic acid microplate method [33]. Fruit total soluble solids and total acidity were measured, as described above.

### 2.8. Determination of Disease and Stay-Green Fruit Indices After Fruit Ripening Phase

At beginning of storage period, fruit shape index was calculated by using following equation:


$$Fruit\;Shape\;Index\;=\frac{Fruit\;\;Length\;(cm)}{Fruit\;\;Diameter\;(cm)}$$

At the fully ripe stage, disease index was calculated according to the following formula [34]:


$$Disease\;index\;=\Sigma \frac{Severity\;\;level\;\;\times \;\;The\;number\;of\;fruits\;at\;this\;level}{highest\;level\;\times Total\;number\;of\;fruits}\;\times 100\%\;$$

The proportion of lesion area is divided into four grades. Zero is grade 0, less than 10% is grade 1, 10~20% is grade 2, 20~50% is grade 3, and greater than 50% is grade 4. In addition, stay-green fruit indices were calculated by counting the number of fruits that failed to undergo normal color change relative to the total number of fruits in the treatment group.

### 2.9. Determination of Metabolite Profiles of Mango Fruit After Fruit Ripening Phase

To evaluate the overall fruit quality responses under different GA_3_ treatments along with control, eleven indicators, i.e., fruit length, fruit diameter, fruit shape index, single-fruit weight, color b-value, fruit firmness, total soluble solids (TSS), vitamin C, total acidity (TA), disease index and weight loss rate, were measured during fruit storage phase. These variables were then subjected to principal component analysis (PCA) to reduce dimensionality and identify the major components contributing to variation among treatments. The variance contribution rate of each principal component and the cumulative contribution rate were used as weighting factors. Using these weights, in combination with the loading matrix and principal component scores, comprehensive scores were calculated for each treatment. Treatments were then ranked according to their comprehensive PCA scores to determine overall quality performance. Based on these integrated PCA results, control and GA100 treatments were selected for subsequent metabolite profiling of mango fruits after 9 days of storage (24 June 2025). Fruit metabolite profiles (volatile organic compounds) of control and GA100 treatments were determined. For this purpose, mango fruits of these treatments were frozen in liquid nitrogen and stored at −80 °C for further analysis. Approximately, 500 mg of frozen tissue sample was ground in liquid nitrogen and transferred to 20-mL headspace vial. Afterwards, 1 mL of saturated sodium chloride (≥99.5%, Sangong Biology, Guangzhou, China) solution was added in the fruit pulp sample. Separately, a 10 μL of normal alkanes mixed standard solution (10 mg L^−1^) was taken into a separate headspace vial. The sample was prepared for gas chromatography-mass spectrometry (GC-MS) analysis. The GC-MS analysis was conducted using head-space solid-phase microextraction pre-treatment, completed through PAL automatic injection system at incubation temperature of 60 °C, preheating for 15 min, extraction for 30 min, and desorption for 4 min. Afterwards, the sample was analyzed using GC-MS (7890B, Agilent, Santa Clara, CA, USA) with a DB-Wax column, with helium as the carrier gas at column flow rate of 1.0 mL min^−1^, injection port temperature of 250 °C and a blowing flow of 3 mL min^−1^. The temperature program conditions were as follows: initial temperature of 40 °C for 4 min, then rising to 245 °C at a rate of 5 °C min^−1^ and maintaining for 5 min. The mass spectrometry interface temperature was 250 °C, the ion source temperature was 230 °C, the quadrupole temperature was 150 °C, the ionization mode was electron impact, with an electron energy of 70 eV. Data acquisition was in full scan mode, with a mass scan range of *m*/*z* 20–400, and a solvent delay of 2.37 min.

### 2.10. Data Analysis

Data were expressed as the mean ± standard error from three replicates. Multiple comparison test was performed by using SPSS statistics software 28, IBM, USA (IBM Corp., Armonk, NY, USA), and differences among mean values were computed based on LSD test at 95% confidence index (*p* ≤ 0.05). Heatmaps of fruit metabolites were drawn by using “pheatmap” packages. After analyzing the mass spectrometry data using Chroma TOF V 4.3x software and the NIST library [35], normalization processing was performed. Log (LOG) transformation and centralization (CTR) formatting were applied to the data using SIMCA V18.0.1 software. Graphs were drawn using Origin 2024 and R software version 4.1.2.

## 3. Results

### 3.1. Fruit Growth During the Expansion Phase

**Figure 1:**
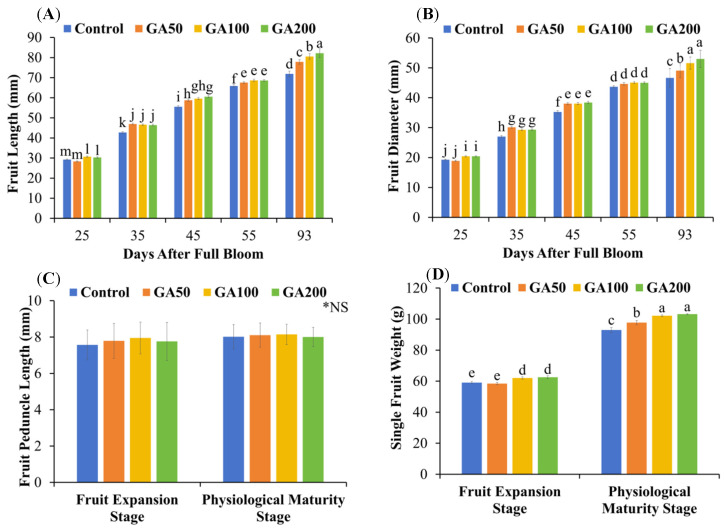
Mango fruit length (**A**), fruit width (**B**), fruit peduncle length (**C**) and single-fruit weight (**D**) at various stages after preharvest application of 50 (GA50), 100 (GA100) and 200 (GA200) mg L^−1^ of gibberellic acid (GA_3_) along with control (foliar application of deionized water). Error bars show standard error of the mean. Fruit expansion and physiological maturity stages reached after 55 and 93 days after full bloom, respectively. Different small letters represent significant differences among treatments at 5% probability level after LSD test. * NS = Non-significant.

Both fruit length and diameter were increased after treatment application (Figure 1A,B). Mean fruit lengths of 80.23 mm and diameter 51.23 mm were measured at physiological maturity (93 days after full bloom (DAFB)), whereas the lowest values of 29.77 mm length and 19.92 mm diameter were observed at 25 DAFB in GA_3_-treated mango. In control, fruit length and diameter reached 71.92 mm and 46.65 mm at physiological maturity, with the lowest values of 29.22 mm and 19.31 mm at 25 DAFB. No significant differences in fruit length and diameter were observed among GA_3_-treated mango 55 DAFB (*p* > 0.05). However, GA50, GA100 and GA200 had 8, 12 and 14% higher fruit length than control (*p* < 0.05). The respective increments in fruit diameter were 5, 11 and 14%. Interestingly, GA200 had 4 and 14% more fruit length and diameter at physiological maturity than other GA_3_ treatments (*p* < 0.05). A strong correlation coefficient between fruit length and diameter was 0.984, indicating strong positive correlation and high synchrony during the fruit expansion phase. Fruit length and diameter were significantly higher in GA100 and GA200 treatments during the whole fruit expansion phase than the control (*p* < 0.05). The mean fruit diameter growth rate was highest (47%) from 25 to 35 DAFB followed by 29% from 35 to 45 DAFB, 19% from 45 to 55 DAFB and only 12% from 55 to 93 DAFB. Similarly, the fruit length growth rate was 54% from 25 to 35 DAFB, 28% from 35 to 45 DAFB, 16% from 45 to 55 DAFB and 15% from 55 to 93 DAFB in all treatments. Overall, fruit length and diameter were increased by 169% and 157% in GA_3_-treated mango from 25 to 93 DAFB, respectively. Their respective increase was 146% and 142% in the control.

No significant differences in fruit peduncle lengths were observed at the both fruit expansion (55 DAFB) and physiological maturity (93 DAFB) stages among GA_3_ treatments (*p* > 0.05; Figure 1C). The mean peduncle lengths of treated fruits were 7.83 and 8.09 mm at 55 and 93 DAFB, respectively. The respective values for the control were 7.57 and 8.02 mm. The mean peduncle length of all treatments was 7.77 and 8.07 mm at the fruit expansion and physiological maturity stages, respectively. Single-fruit weight was significantly (*p* < 0.05) higher in GA100 and GA200 treatments relative to control, while no significant differences were observed between GA50 and control at the fruit expansion stage (Figure 1D). The increment was 5 and 6% higher in GA100 and GA200 than the control at this stage. However, significantly higher weight was measured in all treated fruits compared to control at the physiological maturity stage. The application of GA_3_ enhanced the fruit weight by 5, 10 and 11% in GA50, GA100 and GA200 treatments compared to control at this stage, respectively. The fruit growth was 57, 67, 64 and 65% in control, GA50, GA100 and GA200 from fruit expansion to the physiological maturity stage. The mean fruit weight was 60.52 g at the fruit expansion stage and 99.03 g, with a mean increment of 64% in all treatments (*p* < 0.05).

### 3.2. Fruit Quality During the Expansion Phase

**Figure 2:**
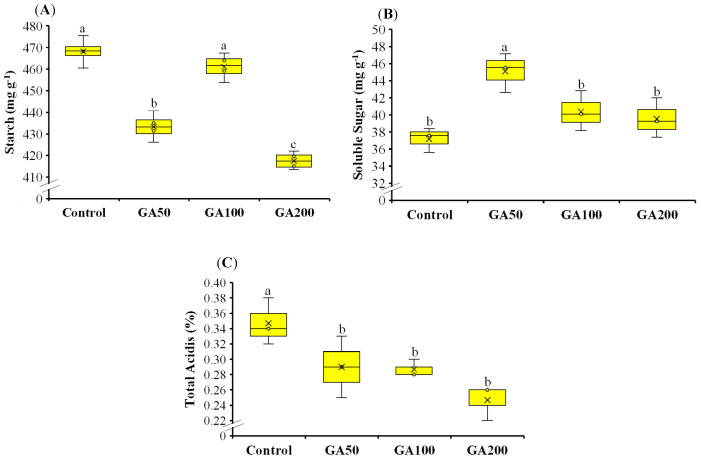
Mango fruit starch (**A**), soluble sugar (**B**) and total acidity (**C**) at fruit expansion stage after preharvest application of 50 (GA50), 100 (GA100) and 200 (GA200) mg L^−1^ of gibberellic acid (GA_3_) along with control (foliar application of deionized water). Different small letters represent significant differences among treatments at 5% probability level after LSD test.

Fruit starch content was reduced by 8, 2 and 11% after application of GA50, GA100 and GA200 compared to the control at the active fruit expansion stage (Figure 2A). Significant differences were observed in GA50 and GA200 treatments, whereas GA100 did not differ significantly from the control. The corresponding starch contents in GA50, GA100 and GA200 were 468, 433, and 461, respectively, compared to 418 mg g^−1^ in the control. The respective values for fruit soluble solids contents were 45.1, 40.4, 39.5 and 37.2 mg g^−1^ (Figure 2B). GA_3_ treatments enhanced the soluble solids by 21, 9 and 6% in GA50, GA100 and GA200 compared to the control. Only GA50 showed a significant increment in fruit soluble sugars compared to the control, while all others did not differ significantly. However, fruit soluble content declined progressively as the GA_3_ application rate increased. Similarly, fruit total acidity declined after the application of GA_3_ (Figure 2C). Fruit acidity was 0.35, 0.29, 0.29 and 0.25% in control, GA50, GA100 and GA200 treatments, respectively. All GA_3_-treated fruits had significantly lower fruit acidity than the control, but the treatments did not differ from each other. The reduction in fruit acidity was 17, 17 and 29% in GA50, GA100 and GA200.

### 3.3. Leaf Antioxidant System During the Fruit Expansion Phase

**Figure 3:**
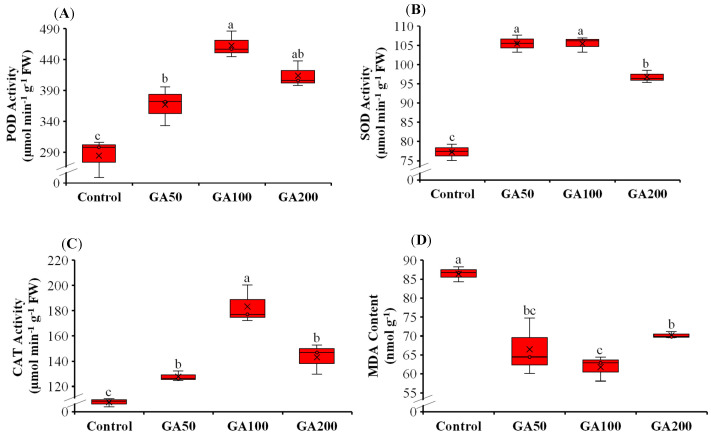
Mango leaf peroxidase (POD) (**A**), superoxide dismutase (SOD) (**B**) and catalase (CAT) (**C**) activities and malondialdehyde (MDA) content (**D**) at fruit expansion stage after preharvest application of 50 (GA50), 100 (GA100) and 200 (GA200) mg L^−1^ of gibberellic acid (GA_3_) along with control (foliar application of deionized water). Different small letters represent significant differences among treatments at 5% probability level after LSD test.

The application of GA_3_ treatment effectively enhanced leaf antioxidant enzyme activities and reduced the degree of membrane lipid peroxidation (Figure 3A–D). Leaf superoxide dismutase (SOD) activity was significantly enhanced in GA_3_-treated plants (Figure 3A). Interestingly, the increase in the application rate of GA_3_ gradually decreased the leaf SOD activity, with the lowest activity observed in GA200 (96.8 µmol min^−1^ g^−1^ FW) compared to GA100 (105.5 µmol min^−1^ g^−1^ FW) and GA50 (105.5 µmol min^−1^ g^−1^ FW). No significant differences in the leaf SOD activity of GA50 and GA100 were observed (*p* > 0.05). Leaf SOD activity was 37, 36 and 25% higher in GA50, GA100 and GA200 than control. Leaf peroxidase (POD) activity was also enhanced by the application of GA_3_ relative to control (Figure 3B; *p* < 0.05). It was highest in GA100 (463 µmol min^−1^ g^−1^ FW), followed by GA200 (414 µmol min^−1^ g^−1^ FW), GA50 (367 µmol min^−1^ g^−1^ FW) and control (284 µmol min^−1^ g^−1^ FW). However, no significant differences were observed between GA50 and GA200 as well as GA100 and GA200 treatments. The increase in leaf POD activity was 29, 63 and 46% in GA50, GA100 and GA200 relative to control, respectively. A similar trend in leaf catalase (CAT) activity was observed, with the maximum value in GA100 (183 µmol min^−1^ g^−1^ FW), followed by GA200 (143 µmol min^−1^ g^−1^ FW), GA50 (128 µmol min^−1^ g^−1^ FW) and control (108 µmol min^−1^ g^−1^ FW) (Figure 3C). This activity was 19, 69 and 32% higher in GA50, GA100 and GA200 than control, respectively (Figure 3D). Interestingly, the leaf malondialdehyde (MDA) content was reduced after the application of GA_3_ treatment, with the lowest value observed in GA100 (61.8 nmol g^−1^), followed by GA50 (66.5 nmol g^−1^), GA200 (70.2 nmol g^−1^) and control (86.4 nmol g^−1^). The leaf MDA activity was 23, 29 and 19% lower in GA50, GA100 and GA200 treatments compared to control, respectively.

### 3.4. Scanning Electron Microscopy of Fruit Pulp

**Figure 4:**
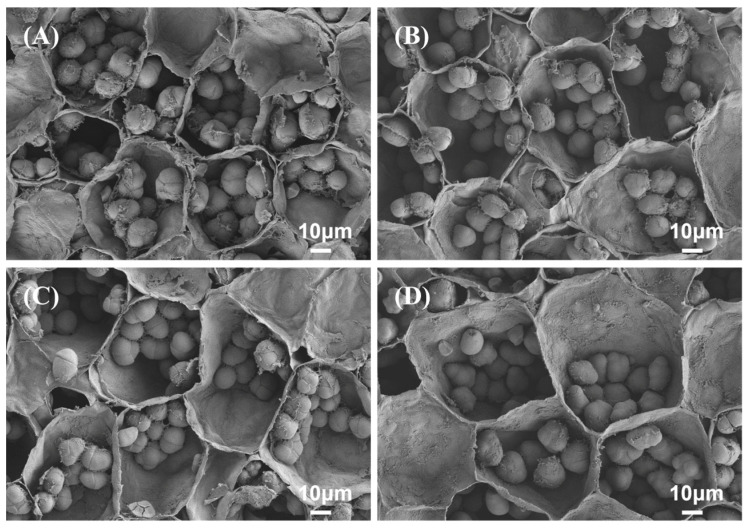
Scanning electron microscope images of mango pulp from control (**A**) and GA3 treatments: GA50 (**B**), GA100 (**C**) and GA200 (**D**), corresponding to preharvest applications of gibberellic acid at rate of 50, 100, and 200 mg L^−1^, respectively.

Scanning electron microscope images of the fruit pulp for each treatment are shown in Figure 4. In each image, the rough spherical structures within the parenchymal cells are starch granules. The diameters of parenchymal cells in GA_3_-treated fruit pulp were larger than control, resulting in a lower number of cells per unit area and indicating a pronounced cell enlargement effect (Figure 4B–D). Cell walls of GA_3_-treated fruit pulp were intact and well-defined, while the control partially collapsed with thinner cell walls, showing tissue softening. Intercellular spaces were small in GA_3_-treated pulp, suggesting more compact and turgid tissue. However, the control had large gaps between cells, indicating cell separation and more water losses than GA_3_-treated mangoes. In addition, treated fruits showed more smoother and uniform surfaces than control, indicating lower cell degradation in these treatments. Additionally, starch granules in GA_3_-treated fruits were prominent and retained their shapes, whereas, in the control, these appeared partially degraded, indicating advance ripening of the fruits. In general, preharvest GA_3_ application enhanced pulp cell integrity, maintained cell structure organization and delayed microstructural deterioration.

### 3.5. Postharvest Physicochemical Changes During Fruit Ripening Phase

**Figure 5:**
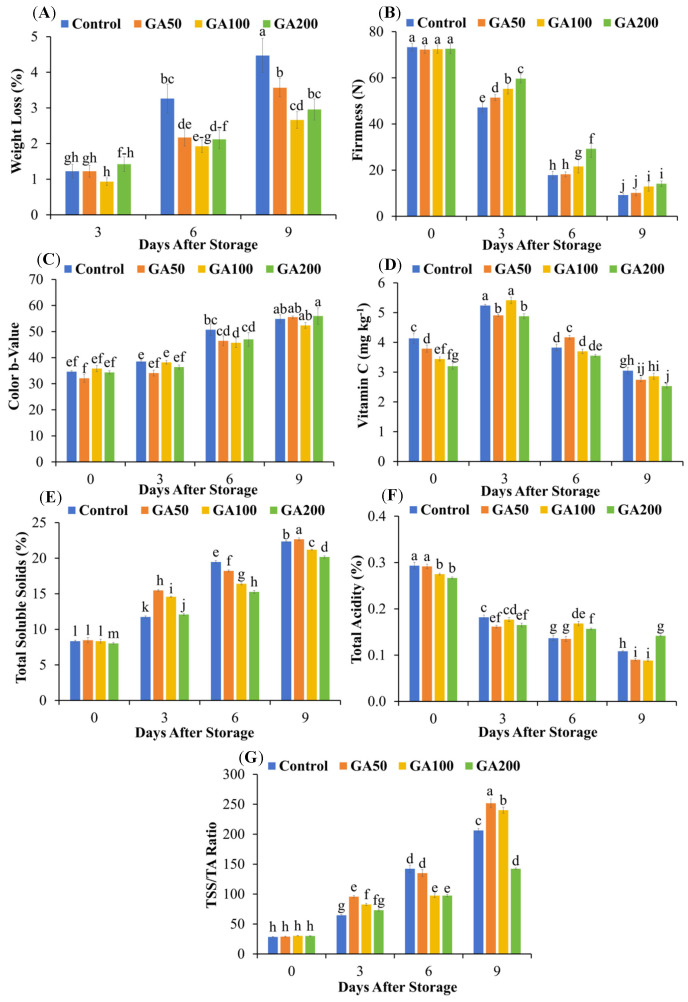
Fruit weight loss (**A**), firmness (**B**), color b-value (**C**), vitamin C (**D**), total soluble solids (TSS; (**E**)), total acidity (TA; (**F**)) and TSS/TA ratio (**G**) during storage after preharvest application of 50 (GA50), 100 (GA100) and 200 (GA200) mg L^−1^ of gibberellic acid (GA_3_) along with control (foliar application of deionized water). Different small letters represent significant differences among treatments at 5% probability level after LSD test.

Fruit weight loss gradually increased during the mango storage period in all treatments (Figure 5A). The mean fruit weight loss rate was 1.2, 2.1 and 3.1% of the initial weight on the third, sixth and ninth days of storage in treated mangoes. Respective values for the control were 1.2, 3.3 and 4.5%. No significant differences in weight loss were observed among treatments after three days of storage. However, weight loss was significantly lower by 33, 42 and 36% in GA50, GA100 and GA200 than control after six days of storage, respectively (*p* < 0.05). The respective decrement was 20, 40 and 33% between six and nine days of storage. On this day, fruit weight losses were 4.5, 3.6, 2.7 and 3.0% in control, GA50, GA100 and GA200, respectively. On average, fruit weight loss increased progressively from 1.2% on day 3 to 2.4% on day 6 and further to 3.4% on day 9 across all treatments (*p* < 0.05).

No differences in fruit firmness were measured at the start of the storage period (*p* > 0.05; Figure 5B). However, as the days passed, fruit firmness in all treatments decreased, but high firmness was maintained in GA_3_-treated fruits compared to the control. The fruit firmness of GA50, GA100 and GA200 was 9, 17 and 27% higher on day 3 of storage than the control. The respective increments were 2, 21 and 64% on days 6, and 11 and 40, and 53% on day 9 of storage. The firmness of mango fruits at full ripening (9 days after storage) increased with the increase in GA_3_ concentrations. At this stage, the control exhibited the lowest firmness (9.18 N), whereas GA200 showed the highest firmness (14.10 N).

At the beginning of the storage period (day 0), the fruit color b-value (yellow–blue axis) did not differ significantly among the control, GA100 and GA200, nor between the control and GA50 (Figure 5C). However, after 3 days of storage, the color b-value did not differ significantly among all treatments. As storage progressed, significant differences in b-values were observed between control and GA100 on the sixth day of the storage period. However, at the full ripening stage, the b-value was again statistically similar among treatments. Over the entire storage period, the color b-value increased by 58% in the control and by 60% in the treated fruits from day 0 to day 9.

Overall, the vitamin C content of mangoes increased initially, reaching the maximum on day 3 of storage, and then declined to its minimum at the full ripening stage (day 9; Figure 5D). Interestingly, the vitamin C content of GA200-treated fruits was lower during the entire storage period than the control. Mean vitamin C contents of treated fruits were 3.5, 5.1, 3.8 and 2.7 mg L^−1^ on 0, 3, 6 and 9 days of storage. The respective values for the control were 4.1, 5.2, 3.8 and 3.0 mg L^−1^. These were 8, 16 and 22% lower in GA50, GA100 and GA200 than control at the start of the storage period (day 0). The respective decrements were 10, 6 and 17% at the end of the storage period (day 9). Only GA50 and GA200 had significantly lower vitamin C content relative to control at the full fruit ripening stage (day 9; *p* < 0.05). At the beginning of the storage period, total soluble solids (TSS) of mango fruits did not differ significantly among control, GA50 and GA100; however, GA200 showed a 4% lower TSS than control (Figure 5E). By day 3 of storage, TSS increased in treated fruits by 32, 24 and 3% in GA50, GA100 and GA200 compared to the control, respectively (*p* < 0.05). On day 6, TSS of the treated fruits decreased by 7, 16 and 22% in GA50, GA100 and GA200 relative to the control (*p* < 0.05). Notably, at the full ripening stage (day 9), TSS was only 1% higher in GA50, while it was 5 and 10% lower in GA100 and GA200 compared to the control, respectively (*p* < 0.05). Overall, the fruit TSS content increased in all treatments over the course of storage. In treated mangoes, TSS increased by 70, 101 and 158% of initial values at day 3, 6 and 9 of storage but in the control by 41, 134 and 168%, respectively.

Fruit total acidity (TA) was gradually reduced by 39, 46 and 61% of the initial value (day 0) on days 3, 6 and 9 of storage in all treatments (Figure 5F). However, at the beginning of storage, no significant differences in fruit total acidity were observed between control and GA50, while it was 6 and 9% lower in GA100 and GA200 than control, respectively. Variations in total acidity occurred during storage. For example, on day 3, fruit total acidity was 11, 3 and 9% lower in GA50, GA100 and GA200 treatments compared to control, respectively. On day 6, it was decreased by only 1% in GA50 while increased by 23 and 15% in GA100 and GA200 than control, respectively. At the full ripening stage (day 9), GA50 and GA100 treatments resulted in 17% and 19% decreases in fruit total acidity compared to control, whereas GA200 treatment caused a 31% increase.

Fruit TSS/TA ratio did not differ at the beginning of the storage period; however, it gradually increased in all treatments during the storage period (Figure 5G). On day 3, the TSS/TA ratio was 48, 28 and 12% higher in GA50, GA100 and GA200 treatments than control, respectively. Similarly, on day 6, respective decrements were 5, 32 and 32%. Interestingly, on day 9, only GA200 maintained a decrement by 31%, while GA50 and GA100 showed increments of 22 and 17%, respectively. Fruit TSS/TA ratio values were 207, 253, 240 and 142 in the control, GA50, GA100 and GA200 at the full ripening stage (day 9), respectively.

### 3.6. Postharvest Changes in Fruit Shape, Disease and Stay-Green Indices During Fruit Ripeni

The application of GA_3_ did not affect fruit shape as no significant differences were observed in the fruit shape index among treatments (*p* > 0.05; Table 1). Moreover, preharvest GA_3_ application effectively delayed the onset of fruit diseases; however, it also negatively affected certain nutritional attributes and contributed to the development of fruit chlorosis. Anthracnose and stem-end rot were the two main postharvest diseases observed during the ripening phase. The disease index was highest (4.60%) in control, followed by GA200 (3.33%), GA100 (2.73%) and GA50 (2.65%) at the end of the ripening phase (Table 1), showing that an increase in the GA_3_ application rate increased the occurrence of disease in fruits. The fruit disease index was 42, 40 and 27% lower in GA50, GA100 and GA200 treatments than control, respectively (Table 1). The fruit color of control and GA50 turned normal, while 2.32 and 7.86% of fruits of GA100 and GA200 stayed green and showed chlorosis at the full ripening stage (Table 1). This rate of chlorotic fruits increased with the increase in the GA_3_ concentration.

### 3.7. Principal Component Analysis of Mango Fruit Physicochemical Attributes

Principal component analysis (PCA) showed that PC1 and PC2 accounted for 61.63 and 85.56% of the cumulative variability among the eleven evaluated fruit quality indicators (Table 2). PC1 had an eigenvalue of 6.779 and contributed 61.63% of the variation. For PC1, fruit physical attributes, including fruit diameter (0.148), fruit firmness (0.146), single-fruit weight (0.144), fruit length (0.143) and weight loss rate (0.131), showed relatively positive loadings. However, fruit biochemical quality attributes exhibited negative loadings on PC1, with fruit TSS (−0.135), fruit vitamin C (−0.127) and fruit total acidity (−0.075) contributing negatively to this component. In addition, color b-value (0.001), disease index (0.081) and fruit shape index (−0.003) had negligible contributions to this component. PC2, which had an eigenvalue of 2.632 and contributed 23.93% of the variance, was characterized by relatively strong positive loadings for fruit total acidity (0.337), fruit shape index (0.308), disease index (0.283), fruit TSS (0.173) and weight loss rate (0.119) but weak positive loading for fruit length (0.058), fruit vitamin C (0.048) and single-fruit weight (0.041). The color b-value (−0.189) showed relatively strong negative loadings on PC2, while fruit firmness (−0.059) and fruit diameter (−0.008) exhibited weak negative loadings. This component reflected differences in chemical composition and postharvest deterioration. Together, these results indicated that PC1 differentiated fruits based on morphological and physical traits, whereas PC2 differentiated those based on chemical quality and susceptibility to diseases.

Evaluation based on PCA scores showed clear differences among treated and untreated fruits. Treatment GA100 achieved the highest comprehensive score (0.56), followed by GA200, GA50 and control (Table 3). The highest comprehensive score of GA100 was attributed to its high positive scores on both PC1 (0.47) and PC2 (0.80). This proved the superior performance of GA100-treated fruits in terms of physical, morphological and chemical quality attributes. In addition, GA200 ranked second, driven by its high positive PC1 score (1.08), suggesting its superior performance in physical and morphological attributes, but the high negative PC2 score (−0.96) suggested its weaker chemical quality and high susceptibility to diseases during mango fruit storage. Contrarily, GA50 obtained a moderate negative PC1 score (−0.30) and high positive PC2 score (0.92), suggesting its poor physical and morphological attributes but high-quality and disease-resistant characteristics. However, both the PC1 (−1.24) and PC2 (−0.76) scores of control treatment were highly negative, indicating the poorest overall fruit physicochemical attributes among all treatments. Based on these results, treatment GA100 was chosen for further metabolite profiling along with control as a check treatment.

### 3.8. Postharvest Metabolomic Changes During Fruit Ripening Phase

**Figure 6:**
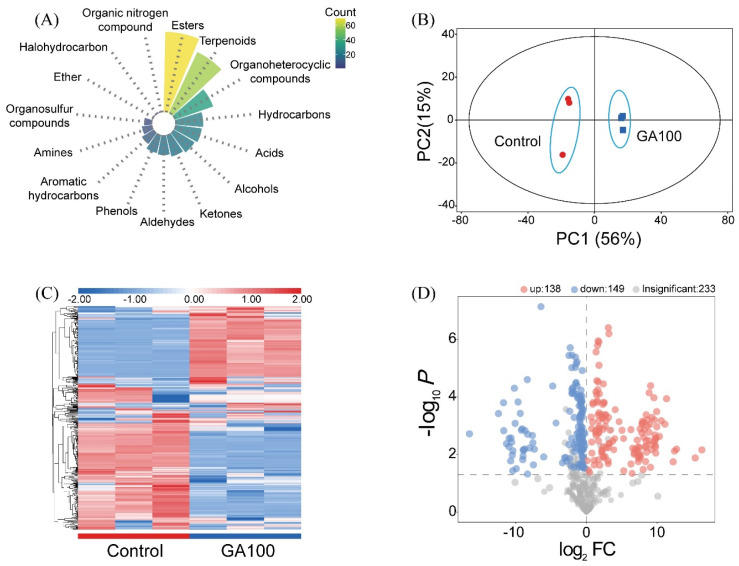
Classification of detected metabolites in mango fruits based on chemical classes (**A**), principal component analysis (PCA) score (**B**), hierarchical clustering heatmap of detected metabolites (**C**) and volcano plot of differentially accumulated metabolites in mango fruit in response to preharvest gibberellic acid application at rate of 100 mg L^−1^ (GA100) relative to control (**D**).

The classification of detected metabolites in mango fruits based on chemical classes is shown in Figure 6A. A total of 520 substances were detected, which showed a complex and diverse metabolic composition for the mango pulp. Among the 520 substances, 418 were qualitatively identified, with 313 being classifiable, while the rest were unclassified. The classifiable compounds were 69 esters, 58 terpenoids, 35 organo-heterocyclic compounds, 24 hydrocarbons, 23 acids, 21 alcohols, 19 ketones, 17 aldehydes, 16 phenols, 12 aromatic hydrocarbons, 9 amines, 7 organosulfur compounds, 1 ether, 1 halo-hydrocarbon and 1 organic nitrogen compound.

Principal component analysis (PCA) was used to evaluate the metabolic differences between control and GA100 (Figure 6B). PC1 and PC2 accounted for 71% of the overall metabolic variation. PC1 showed 56% variation, while PC2 showed only 15%. Significant differences in metabolites were observed between the control and GA100 treatments, with distinct clustering among samples. A clear separation between these two treatments was observed, primarily along PC1, indicating pronounced treatment-induced metabolic differences. Control samples clustered on the negative side of PC1, whereas GA100-treated mango samples were distinctly positioned on the positive side. This showed that GA100 altered the abundance of multiple metabolic compounds in mango fruit. The relatively tight clustering of replicates within each group showed good analytical reproducibility and low variability among replicates. In addition, there were minimal ellipses between the two treatments that confirmed robust metabolic differences.

After normalization of all metabolite data, cluster heatmap analysis was performed (Figure 6C). The heatmap showed clear separation of samples due to treatment effects, indicating that preharvest application of 100 mg L^−1^ GA_3_ (GA100) significantly altered metabolite accumulation compared to control. Several metabolites were regulated in GA100-treated mango fruits, showing a treatment-specific modulation in both primary and secondary metabolites. These patterns suggested that GA100 influenced multiple metabolic pathways, leading to a coordinated shift in the fruit metabolomic profile. Metabolic shifts showed that GA100 induced changes in fruit physiology, potentially affecting ripening, sugar accumulation, secondary metabolites and stress-related compounds.

A total of 520 differential metabolites were screened between control and GA100 treatments using the criteria of variable importance in projection (VIP) > 1 (VIP values derived from the orthogonal partial least squares-discriminant analysis (OPLS-DA) model for group comparison) and *p*-value < 0.05. Among these, 138 were significantly upregulated and 149 were significantly downregulated in GA100-treated mangoes compared to control, based on VIP > 1 and *p* < 0.05, while 233 metabolites were not significantly altered (Figure 6D).

The top 20 differential metabolites were screened, belonging to six major chemical classes, including esters (2), terpenoids (6), alcohols (4), ketones (2), aldehydes (3) and aromatic hydrocarbons (3) (Table 4). Both top identified esters (Ethyle tiglate and Ethyl acetate) exhibited upregulation in GA100-treated fruits compared to control. In contrast, all terpenoids showed a consistent downregulation in GA100 relative to control. These terpenoids included Geraniol, Linalyl isobutyrate, α-Phellandrene, β-Myrcene, D-Limonene and α-Pinene, which demonstrated decreased expression levels in GA100 compared to control. Alcohols displayed mixed expression, with Methyl alcohol and 1-hexanol showing reduced expression, while 1-Butanol and Benzyl alcohol were upregulated in GA100-treated mango fruits. Similarly, ketone Acetoin exhibited increased expression, while 2,3-Pentanedione was downregulated. All top aldehydes (butanal, heptanal, and nonanal) were downregulated in GA100, indicating a general suppression of these compounds. In contrast, all top screened aromatic hydrocarbons, including Styrene, Ethylbenzene and p-Xylene, showed increased expression levels in GA100 compared to control, suggesting an overall enhancement in these compounds.

### 3.9. Postharvest Flavor-Associated Metabolomic Changes During Fruit Ripening Phase

**Figure 7:**
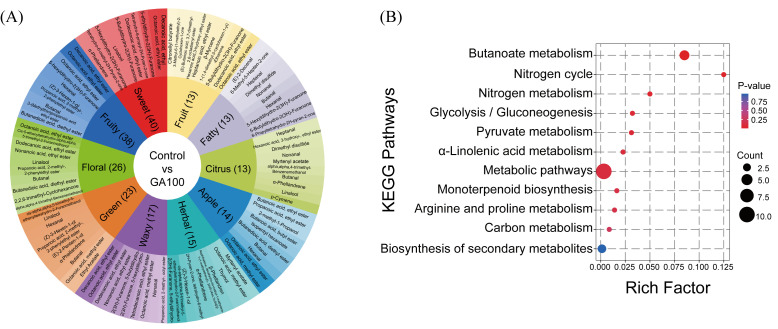
Changes in flavor-associated metabolites after preharvest gibberellic acid application at rate of 100 mg L^−1^ (GA100) relative to control (**A**) and KEGG enrichment diagram of differential metabolites (**B**).

Preharvest application of gibberellic acid at 100 mg L^−1^ (GA100) significantly altered flavor-associated metabolite compounds relative to control, as presented in the mango flavor wheel (Figure 7A). The middle circle represents the top 10 flavor characteristics with the largest number of flavor-associated metabolites, where the numbers in parentheses indicate the count of differential metabolites. The outermost circle shows the differential metabolites corresponding to each flavor characteristic, with the top 10 significant differential metabolites selected based on *p*-values. The background color intensity of differential metabolites indicates the significance of the *p*-value, with darker colors representing more significant *p*-values. These were distributed across sweet (40 metabolites), fruity (38), floral (26), green (23), waxy (17), herbal (15), apple (14), citrus (13), fatty (13) and fruit (13). Notably, sweet, fruity and floral metabolites represented major proportions of the top 10 selected flavor-associated metabolites. In contrast, fewer metabolites were associated with citrus, fatty and fruity tastes in GA100 than control.

### 3.10. Resource-Related Metabolic Pathways

KEGG pathway enrichment analysis of differential metabolites between control and GA100-treated mango fruit revealed significant alterations in various resource-related metabolic pathways (Figure 7B). Enriched pathways were mainly associated with nitrogen cycle, butanoate metabolism, nitrogen metabolism, glycolysis/gluconeogenesis, pyruvate metabolism, α-linolenic acid metabolism, metabolic pathways, monoterpenoid biosynthesis, arginine and proline metabolism as well as carbon metabolism. This indicated extensive reprogramming of central carbon, nitrogen, and lipid metabolisms after preharvest application GA100 treatment. The Rich Factor, defined as the ratio of differential metabolites annotated to a given pathway relative to the total metabolites in that pathway, changed across pathways, with nitrogen cycle and butanoate metabolism showing relatively higher enrichment. The size of the bubbles reflected the number of differential metabolites involved in each pathway; metabolic pathways had the largest number of enriched metabolites (10), followed by butanoate metabolism (4) and biosynthesis of secondary metabolites (3), while the remaining contained only one differential metabolite pathway each. The color gradient represented pathway significance, with deeper red indicating lower *p*-values and higher enrichment significance. Among all enriched pathways, biosynthesis of secondary metabolites was the only pathway shown in blue, reflecting the highest *p*-value and lowest enrichment significance. Overall, these results indicated that the application of GA100 substantially affected the interconnected metabolic networks that underpin flavor formation and physiological metabolism in mango fruit.

## 4. Discussion

### 4.1. Fruit Growth During the Expansion Phase

The application of GA_3_ markedly stimulated mango fruit growth by increasing fruit length, diameter and single-fruit weight (Figure 1), primarily through stimulating cell division and cell elongation in developing tissues. The enhanced fruit size and weight observed in GA_3_-treated mangoes can be attributed to the strengthened sink capacity, higher assimilate accumulation and delayed senescence of fruit tissues [36,37]. Moreover, GA_3_ modulates the endogenous hormonal balance by reducing the influence of growth-delaying hormones and elevating growth-promoting GA, auxin and cytokinin, thereby sustaining rapid fruit enlargement during critical stages of development [38]. Exogenous application of GA_3_ in different mango cultivars (Amrapali, Dashehari, Banganpalli, Naomi, Tommy Atkins, Keitt) has consistently increased fruit length, diameter, individual fruit weight, pulp percentage and overall yield compared with untreated controls [25,39,40]. Transcriptome analysis of GA_3_-treated mango fruit has identified differentially expressed genes associated with hormone signaling and cell wall loosening, which provides mechanistic support for GA_3_-induced fruit expansions [41]. In ‘Tainong’ mango, foliar application of GA_3_ at a rate of 50 mg L^−1^ at the late flowering stage significantly promoted parthenocarpic fruit enlargement and increased single-fruit weight from the second to fifth week after treatment, ultimately improving yield [6]. Similar observations have also been reported in young kumquat fruit, where exogenous application of GA_3_ at 20 mg L^−1^ promoted fruit growth, resulting in increases in both longitudinal and transverse diameters during the fruit expansion phase [42]. In line with these findings, all GA_3_ treatments in our study enhanced young fruit length and diameter and increased single-fruit weight, whereas fruit stalk length remained unaffected (Figure 1A–C). The lack of response in fruit stalk length suggests that GA_3_ activity was largely restricted to fruit tissues rather than peduncle, consistent with evidence that gibberellins preferentially stimulate cell elongation and expansion in actively growing sink organs rather than uniformly affecting associated supporting plant structures [43]. Comparable organ-specific responses have been described in grapes, where GA_3_ increased berry size but had a limited effect on pedicel length, mainly altering its thickness and mechanical strength [44]. Overall, these findings indicate that GA_3_ sprays applied shortly after full bloom are an effective strategy to enhance mango fruit size, individual fruit weight and, consequently, improve marketable yield. These developments are commercially important, as larger and firmer fruits are more attractive to consumers and better withstand handling and transportation, thereby mitigating postharvest losses.

### 4.2. Fruit Quality During the Expansion Phase

Preharvest application of GA_3_ shifted carbohydrate metabolism toward a more advanced ripening profile, as depicted by reduced starch and total acidity (up to 11% and 29%, respectively) while improving soluble sugar content (up to 21%) during the fruit expansion phase (Figure 2A–C). This indicated that preharvest application of GA_3_ may promote the conversion of stored starch into soluble sugar and enhance the dilution of organic acids, leading to a high sugar-to-acid ratio and, consequently, improving fruit sweetness. Similarly, application of GA_3_ in grapes and date palm enhanced fruit sugar content while reducing their titratable acidity [36,45]. This might be attributed to the stimulated activity of starch-degrading enzymes and their gene expression, which affected carbohydrate transformation and distribution, ultimately contributing to ripening-associated carbon remobilization. Foliar application of GA_3_ on ‘Tainong’ mangoes has been reported to increase fruit soluble sugar content, indicating enhanced conversion of starch to soluble sugars [46,47]. In addition, foliar application of GA_3_ has been reported to reduce citric and malic acid contents in ‘Newhall’ navel oranges during the fruit development phase, resulting in decreased total acidity and an increased sugar-to-acid ratio [46]. In our study, GA_3_ application increased fruit soluble sugar content while decreasing starch and total acidity levels during the fruit development phase, which is consistent with the literature. From a production viewpoint, these changes may enhance fruit sweetness without excessive acid retention, which could improve taste at harvest and better align with consumer preferences.

### 4.3. Leaf Antioxidant System During the Fruit Expansion Phase

Preharvest foliar application of GA_3_ markedly increased the activities of key antioxidant enzymes (SOD, POD and CAT) in mango leaves while simultaneously reducing the MDA content. This shows that GA_3_ strengthened the plant antioxidant defense system and mitigated lipid peroxidation, which is a major indicator of cellular damage. Such modulation of the leaf redox status suggests that GA_3_ application might enhance the ability of plants to detoxify reactive oxygen species (ROS), thereby alleviating oxidative damage caused by abiotic stresses during the fruit expansion phase [48,49]. Similar GA_3_-driven regulation of redox homeostasis has been reported in multiple crops. For example, foliar application of GA_3_ alleviated Cu toxicity in peas by stimulating the activities of SOD, POD and CAT while reducing MDA and H_2_O_2_ levels in the leaves [50]. In addition, exogenous application of GA_3_ enhanced photosynthetic activity of maize under low-light conditions by activating the same enzymes and reducing MDA and H_2_O_2_ levels in the leaves [51]. Similar positive effects of GA_3_ have been reported in tomato under heat stress, Brassica juncea and maize exposed to salinity and cadmium [51,52,53,54]. Foliar application of GA_3_ at a rate of 20 to 50 mg L^−1^ on ‘Newhall’ navel orange seedlings increased leaf SOD, POD, and CAT activities while reducing cell membrane permeability, suggesting that GA_3_ enhances cellular stability by activating the antioxidant enzyme system [46]. In banana plants under high-temperature stress, foliar application of GA_3_ at a rate of 50 mg L^−1^ reduced leaf MDA content and increased SOD and CAT activities [55].

In our study, a clear dose-dependent effect of GA_3_ application on leaf antioxidant system during the fruit expansion phase was observed (Figure 3). Application of GA_3_ at 50 (GA50) and 100 (GA100) mg L^−1^ enhanced antioxidant enzyme activities in mango leaves with the concomitant synthesis of protective compounds like L-proline and terpenes that may have maintained cellular osmotic balance and contributed in scavenging the reactive oxygen species (ROS). Consequently, this antioxidant system supports photosynthesis by safeguarding cellular membranes, chloroplast function and mesophyll conductance, enhancing CO_2_ diffusion to carboxylation sites, thereby promoting carbon assimilation and energy metabolism in the leaves. Contrarily, a high concentration of 200 (GA200) mg L^−1^ caused oxidative damage, lipid peroxidation (evidenced by increased MDA content; Figure 3D), and the disruption of metabolic pathways, leading to reduced photosynthesis and impaired growth. This biphasic response is consistent with a hormetic effect of GA_3_, whereby low to moderate doses (GA50 and GA100) activate redox-sensitive signaling pathways and antioxidant defenses. However, excessive GA_3_ (GA200) induces oxidative stress and physiological disruption [56,57]. Such dose-dependent modulation of redox homeostasis by GA_3_ aligns with broader principles of redox biology and has been reported in spinach, peas and wheat [50,58,59]. In contrast, fruits often respond differently to GA_3_ doses, with high application rates often delaying ripening processes, partly through the modulation of antioxidant systems and hormone signaling such as ethylene and abscisic acid (ABA), thereby affecting metabolic shifts such as pigment accumulation and cell wall modification [16,58]. In kiwifruit, GA_3_ application suppressed ethylene production and cell wall modification genes, thereby delaying softening and ripening [59]. Mechanistically, leaves prioritize the maintenance of photosynthetic capacity and cellular integrity under GA_3_ application, whereas fruits balance antioxidant defenses with ripening-associated oxidative signaling, which drives quality traits such as color, flavor, and texture, reflecting distinct metabolic and hormonal sensitivities [60,61]. Therefore, defining an optimum GA_3_ dose for fruits like mango is critical as excessive application may shift its effect from protective to damaging in leaves, while in fruits, it may become ripening modulating or even ripening delaying, highlighting the dose-dependent and tissue-specific nature of GA_3_ responses. Factors such as leaf age and light exposure can significantly affect the antioxidant enzyme system [60,61]; therefore, we suggest that temporal sampling of leaves in synchrony with fruit development stages may better elucidate the effects of preharvest GA_3_ applications on source–sink relationships and fruit physicochemical attributes.

### 4.4. Postharvest Physiochemical Changes During Fruit Ripening Phase

In our study, GA_3_-treated fruits exhibited decreased weight loss, enhanced firmness, more uniform coloration and reduced disease incidence compared with the control (Figure 5A–C). Similar reductions in physiological weight loss and maintenance of fruit firmness following pre- and postharvest GA_3_ applications have been reported in mango, where dipping or spraying at a rate of 100 to 400 mg L^−1^ significantly decreased weight loss and extended shelf life compared with untreated fruits [15,16,62]. Lower weight loss observed in GA_3_-treated fruits indicated that its preharvest foliar application effectively reduced transpiration and respiration losses during the fruit ripening phase. It is extensively reported that GA_3_ lowers metabolic activity, reduces tissue permeability, slows water loss and delays ripening in mango and other climacteric fruits [15,63]. Firmness retention is associated with GA_3_-mediated suppression of ethylene production and cell wall degrading enzymes, resulting in slower degradation of structural polysaccharides and better maintenance of cell integrity [64]. Similar GA_3_-induced maintenance of fruit firmness has been reported in mango, grapes, and cherry fruits [15,29,63] primarily due to maintenance of cell wall integrity via calcium retention and lignification, which contributes to enhanced resistance against physiological breakdown and decay [29]. Lower fruit weight loss and firmer texture observed in GA_3_-treated mangoes contributed to a reduced incidence of diseases, as intact and turgid tissues were less susceptible to pathogen invasion (Table 1). Application of GA_3_ reduced postharvest diseases in peach fruits by enhancing antioxidant enzyme activities, upregulating antioxidant-related genes, and suppressing ethylene production and respiration rate [65]. Similarly, GA_3_ lowers disease incidence in cherries [66]. In our study, disease incidence increased at high GA_3_ concentration (GA200) because excessive GA_3_ can disrupt normal fruit physiology, leading to delayed ripening and incomplete coloration, as indicated by a high stay-green fruit index of 7.86. These conditions favor pathogen development, resulting in a disease index of 3.33%. In contrast, the GA100 treatment exhibited lower stay-green indices (2.32%) along with more uniform color development and synchronized ripening compared to the other treatments (Table 1). Additionally, combined applications of GA_3_ (25–50 mg L^−1^) and N-(2-Chloro-4-pyridyl)-N′-phenylurea (2 mg L^−1^) after flowering have been shown to optimize fruit shape and coloration in ‘Hutai No. 8’ and ‘Summer Black’ grapes, thereby enhancing overall fruit quality. Therefore, moderate GA_3_ concentrations are crucial to balance the benefits of delayed ripening with disease resistance and uniform fruit maturation. Overall, the firmer, heavier, and disease-free ripe mango fruits are more appealing to consumers and better withstand handling and transportation damages, thereby enhancing marketability and increasing farmers’ profit. The lower vitamin C in GA_3_-treated fruits at the ripening stage compared with the control may be attributed to increased oxidative and respiratory utilization of ascorbic acid during the extended ripening period. Ascorbic acid may act as a major non-enzymatic antioxidant and respiratory substrate over an extended period, leading to its greater depletion by the end of ripening phase [67]. The effect was more pronounced at higher GA_3_ dose (GA200), which resulted in the highest stay-green index (7.86%; Table 1), indicating the maximum delay in ripening and senescence, and consequently leading to intensified ascorbic acid consumption during the ripening process. However, contrasting results have been reported in the literature where GA_3_ application was found to enhance fruit vitamin C content [68,69]. The amount of vitamin C retained during storage period appears to depend on both the GA_3_ dose and the storage temperature. For example, Talat et al. and Cai et al. [53,54] applied GA_3_ at 20–45 mg L^−1^ and stored fruit at 6–25 °C, whereas in our study GA_3_ rates were 50–200 mg L^−1^ and the fruits were stored at room temperature. Interestingly, we also observed that GA_3_ slowed the increase in total soluble solids (TSS) in mango fruits during the ripening phase which may be attributed to reduced amylase activity, directly limiting the hydrolysis of stored starch into soluble sugars. Amylase activity is generally reported to increase during the early stages of ripening, promoting starch degradation and sugar accumulation, thereby contributing to an increase in TSS. Total acidity (TA) exhibited a dose-dependent response to GA_3_ application, with the highest concentration (200 mg L^−1^; GA200) retaining more TA compared to the lower and medium concentrations (50–100 mg L^−1^; GA50-GA100; Figure 5F). This effect likely occurs because high GA_3_ dose delayed fruit ripening, as evidenced by the stay green data, thereby slowing the breakdown of organic acids and maintaining higher fruit TA. Contrarily, TSS/TA ratio decreased at the higher level GA_3_ level (GA200) while increased at lower to medium levels (GA50-GA100; Figure 5G). At GA200, higher TA combined with a relatively lower TSS resulted in a reduced TSS/TA ratio, indicating a more acidic fruit profile. Conversely, GA50 and GA100 treatments tended to reduce TA while increasing TSS, resulting in a higher TSS/TA ratio, indicative of sweeter fruit quality, which may align well with consumer flavor preferences [15].

### 4.5. Postharvest Metabolomic Changes During Fruit Ripening Phase

Mango fruit aroma is primarily determined by its volatile compounds, which not only indicate fruit ripeness but also serves as key indicators of commercial quality. In our study, metabolomics analysis identified 287 differentially regulated metabolites between GA100 and the control (Figure 6D). Xin et al. [70] reported 181 volatile compounds across different developmental and storage stages of ‘Tainong 1’ mangoes, including terpenoids (e.g., α-pinene), aldehydes (e.g., (E, Z)-2,6-nonadienal), and alcohols (e.g., Geraniol), among which ethanol and (E, Z)-2,6-nonadienal were identified as key aroma-active compounds. In our study, mango pulp contained 313 identifiable volatile compounds at ripening stage (Figure 6A). Li et al. [71] demonstrated that the characteristic aroma of fresh ‘Keitt’ mangoes is primarily determined by 11 key aroma-active compounds, including terpenes (e.g., β-myrcene, β-caryophyllene), aldehydes (e.g., hexanal, nonanal), and esters (e.g., ethyl acetate), which play decisive roles in producing the distinctive grassy, woody, and citrus-like fruity notes. The pronounced grassy and pine-resin aromas of ‘Tainong’ mangoes is primarily attributed to their high content of terpenoids particularly β-ocimene and limonene. These compounds are characteristic flavor markers of ‘Tainong’ mangoes, whereas the excessive accumulation of propyl butyrate during late storage negatively impairs fruit quality [72]. In our study, application of GA_3_ at a rate of 100 mg L^−1^ (GA100) markedly altered the volatile profile by suppressing the biosynthesis of terpenoids (Geraniol, Linalyl isobutyrate, α-Phellandrene, β-Myrcene, D-Limonene and α-Pinene) as well as several aldehydes (Butanal, Heptanal and Nonanal) and alcohols (Methyl alcohol and1-Hexanol) that contribute to floral, fruity, green, and citrus notes (Table 4). In parallel, GA100 stimulated the accumulation of selected esters (Ethyl acetate and Ethyl tiglate) and ketones (Acetoin), which primarily impart simple fruity, sweet, and creamy attributes. Although these compounds increased, they were insufficient to compensate for the reduction in terpenoid-derived volatiles, resulting in a less complex and more uniform aroma profile at full ripening in GA100-treated fruits. Overall, GA100 appears to simplify mango aroma by favoring sweet, ester-driven notes at the expense of floral and green nuances, indicating that it reshapes rather than enhances aroma complexity. In conclusion, GA_3_ application represents a promising strategy to extend mango shelf life and modulate aroma development; however, it tends to simplify the aroma profile by favoring ester production at the expense of complex terpenoid-derived notes. Improving shelf life significantly extends time for mango fruit transportation, handling and postharvest storage, thereby enhancing overall marketability by reducing losses and preserving postharvest quality during distribution. However, the simplified aroma profile may adversely affect sensory quality, particularly aroma complexity, which can vary across different regions of the world. For example, consumers in South Asia, such as in Pakistan and India, often prefer sweeter mangoes, whereas consumers in other regions, such as Indonesia and China, tend to favor a balance of sour and sweet flavors [73]. Therefore, despite the potential trade-off in aroma profile, the physical improvements in firmness, weight retention, and disease resistance in mango fruits after the ripening phase contribute to higher commercial value by ensuring more consistent fruit quality and extended shelf life needed for fruit transportation.

### 4.6. Resource-Related Metabolic Pathways

KEGG pathway enrichment analysis revealed that butanoate metabolism and monoterpenoid biosynthesis are the key metabolic pathways underlying the flavor differences between GA_3_-treated and control mangoes. The butanoate metabolism pathway showed the highest level of enrichment (Figure 7B). Butyric acid and its derived esters (e.g., ethyl butyrate) are important aroma compounds in fruits, typically contributing sweet, fruity, and wine-like notes [74,75,76]. This indicated that aroma differences between control and GA100 are attributed to the metabolic regulation of short-chain fatty acids and their esters. Additionally, the enrichment of monoterpenoid biosynthesis, including compounds such as D-limonene and linalool, contributes to the floral, pine, and citrus aroma characteristics of fruits like mangoes and citrus. Differences in the synthesis and metabolism of terpenoids between control and GA100-treated mangoes represent the core biological process driving the development of unique flavors. In addition, variation in nitrogen and arginine and proline metabolisms may indirectly regulate the production of aroma compounds such as alcohols, aldehydes, and esters by modulating the pool of amino acids that serve as precursors for these volatiles. Also, α-linolenic acid metabolism led to the formation of fatty acid-derived volatiles, such as C6 aldehydes and alcohols, indicating that lipid degradation contributes to aroma development. KEGG pathway enrichment analysis indicated that GA100 application affected carbon and nitrogen metabolism in mango fruits; however, these effects are complex and not directly causal. GA100 treatment may enhance the activities of key enzymes involved in carbon and nitrogen metabolism, including invertase, amylase and nitrate reductase, which could collectively contribute to improved fruit quality [77]. These metabolic adjustments suggest that GA_3_ modulates carbohydrate and nitrogen utilization pathways; however, KEGG pathway enrichment alone does not establish direct causal links between GA_3_ application and specific metabolic changes. GA_3_ functions as a regulator that enhances metabolic processes related to carbon and nitrogen, but the precise molecular mechanisms and causal pathways require further research beyond KEGG pathway enrichment analysis. Overall, these results indicate that the distinct aroma profile in GA100-treated mangoes was not governed by a single metabolic pathway but, rather, by a combination of synergism between the pathways dominated by butanoate metabolism, monoterpenoid biosynthesis, amino acid metabolism, and fatty acid degradation.

## 5. Conclusions

This study concluded that preharvest application of gibberellic acid (GA_3_) exerts dose-dependent effects on mango, influencing fruit growth, physicochemical attributes and the leaf antioxidant system during the fruit expansion phase, while also modifying physical, visual, biochemical, and postharvest quality traits during fruit ripening. Principal component analysis (PCA) was employed to determine the optimal GA_3_ dose, with 100 mg L^−1^ (GA100) identified as the most effective treatment. Metabolite profiling identified a total of 520 compounds, highlighting the complex and diverse metabolic composition of mango pulp. Further metabolomics analysis identified 287 differentially accumulated metabolites between GA100 and the control, with the top 20 belonging to six major chemical classes (esters, terpenoids, alcohols, ketones, aldehydes, and aromatic hydrocarbons), which exhibited a strong response to GA100. Treatment GA100 appeared to simplify mango aroma by promoting sweet, ester-driven notes at the expense of terpenoid-derived floral and green nuances, suggesting that it reshapes rather than enhances aroma complexity. This distinct aroma profile was associated not with major alterations in specific aroma biosynthetic pathways but with changes in broader resource-related pathways, including central carbon (glycolysis/gluconeogenesis, pyruvate metabolism, butanoate metabolism), nitrogen (nitrogen cycle, arginine and proline metabolism), lipid (α-linolenic acid metabolism), and secondary (monoterpenoid biosynthesis) metabolisms, which collectively contributed to the modified aroma profile. Overall, preharvest application of GA_3_ at 100 mg L^−1^ is recommended to enhance mango fruit growth, physicochemical attributes, and shelf life, although it alters the characteristic aroma of ‘Tainong 1’ from floral and green notes toward sweeter and more fruity notes. In addition, this study was conducted over a single year, which may limit the generalizability of the results to regions with different climatic conditions. Future studies should standardize fruit sampling based on consistent physiological maturity with similar ripening indicators to establish more precise and absolute comparisons of fruit physicochemical attributes.

## Figures and Tables

**Table 1:** Mango fruit shape, disease and stay-green indices during fruit ripening stage.

Treatment	Shape Index	Disease Index (%)	Disease Name	Stay-Green Fruit Incidence (%)
Control	1.55 ± 0.11 a	4.60 ± 0.19 a	Anthracnose and stem-end rot	/ #
GA50 *	1.59 ± 0.08 a	2.65 ± 0.17 c	-do-	/
GA100	1.56 ± 0.07 a	2.73 ± 0.32 c	-do-	2.32 ± 0.14 b
GA200	1.56 ± 0.09 a	3.33 ± 0.41 b	-do-	7.86 ± 0.34 a

# Not applicable; * GA50, GA100 and GA200 correspond to preharvest applications of gibberellic acid at a rate of 50, 100, and 200 mg L^−1^, respectively. Different small letters as superscript within a column represent significant differences at 5% probability level.

**Table 2:** Principal component loadings, eigenvalues and variance contribution rates of mango fruit quality indicators.

Indicators	Load	
	PC1	PC2
Fruit Length	0.143	0.058
Fruit Diameter	0.148	−0.008
Single-fruit weight	0.144	0.041
Weight Loss Rate	0.131	0.119
Fruit Firmness	0.146	−0.059
Color b-value	0.001	−0.189
Fruit Vitamin C	−0.127	0.048
Fruit TSS *	−0.135	0.173
Fruit Total Acidity	−0.075	0.337
Fruit shape index	−0.003	0.308
Disease Index	0.081	0.283
Eigenvalue	6.779	2.632
Variance Contribution Rate (%)	61.63	23.93
Cumulative Variance Contribution Rate (%)	61.63	85.56

* TSS = Total Soluble Solids.

**Table 3:** Principal component evaluation and comprehensive ranking of mango fruit quality under different gibberellic acid (GA_3_) treatments.

Treatment	Principal Component Score		Comprehensive Score	Comprehensive Ranking
	PC1	PC2		
Control	−1.23774	−0.76251	−1.10	4
GA50 *	−0.30405	0.9236	0.04	3
GA100	0.46514	0.80052	0.56	1
GA200	1.07666	−0.96161	0.51	2

* GA50, GA100 and GA200 correspond to preharvest applications of gibberellic acid at a rate of 50, 100, and 200 mg L^−1^, respectively.

**Table 4:** Expression level of key metabolites affected by the application of gibberellic acid.

No.	Compound Name *	Molecular Formula	Class	Expression Level ∓
1	Ethyl tiglate	C_7_H_12_O_2_	Esters	↑
2	Ethyl acetate	C_4_H_8_O_2_	Esters	↑
3	Geraniol	C_10_H_18_O	Terpenoids	↓
4	Linalyl isobutyrate	C_14_H_24_O_2_	Terpenoids	↓
5	α-Phellandrene	C_10_H_16_	Terpenoids	↓
6	β-Myrcene	C_10_H_16_	Terpenoids	↓
7	D-Limonene	C_10_H_16_	Terpenoids	↓
8	α-Pinene	C_10_H_16_	Terpenoids	↓
9	Methyl alcohol	CH_4_O	Alcohols	↓
10	1-Hexanol	C_6_H_14_O	Alcohols	↓
11	1-Butanol	C_4_H_10_O	Alcohols	↑
12	Benzyl alcohol	C_7_H_8_O	Alcohols	↑
13	Acetoin	C_4_H_8_O_2_	Ketones	↑
14	2,3-Pentanedione	C_5_H_8_O_2_	Ketones	↓
15	Butanal	C_4_H_8_O	Aldehydes	↓
16	Heptanal	C_7_H_14_O	Aldehydes	↓
17	Nonanal	C_9_H_18_O	Aldehydes	↓
18	Styrene	C_8_H_8_	Aromatic hydrocarbons	↑
19	Ethylbenzene	C_8_H_10_	Aromatic hydrocarbons	↑
20	p-Xylene	C_8_H_10_	Aromatic hydrocarbons	↑

* Filtered under the criteria of VIP > 1, *p*-value < 0.01, and Fold-Change > 2; ∓ The upward and downward arrows show upregulation and downregulation of metabolites, respectively.

## Data Availability

The original contributions presented in this study are included in the article. Further inquiries can be directed to the corresponding authors.
